# Clobazam and Valproate, but Lamotrigine, a Sodium Channel Inhibitor, Reduce the Incidence of Hyperthermia‐Induced Clonic Seizures in Dravet Syndrome Mice: Assessment of Anti‐Seizure Effects in Mice Using a Stabilized Ambient Temperature System

**DOI:** 10.1002/npr2.70168

**Published:** 2026-09-15

**Authors:** Futa Sato, Mayuka Nakamura, Megumi Oishi, Yuki Matsuo, Naoki Amada, Takashi Wadatsu, Nobuyoshi Jinnai, Yusuke Kakumoto, Kenji Maeda, Yuki Yamasaki, Takashi Futamura

**Affiliations:** ^1^ Department of CNS Research Otsuka Pharmaceutical Co. Ltd. Tokushima Japan; ^2^ Department of Lead Discovery Research Otsuka Pharmaceutical Co. Ltd. Tokushima Japan; ^3^ Medical Affairs Department Otsuka Pharmaceutical Co. Ltd. Tokyo Japan

**Keywords:** clobazam, epilepsy, hyperthermia, lamotrigine, seizures, valproic acid

## Abstract

**Background:**

Dravet syndrome (DS) is a severe and treatment‐resistant epileptic encephalopathy, most commonly caused by de novo mutations in the sodium voltage‐gated channel alpha subunit 1 (*SCN1A*) gene. Patients with DS often present with febrile seizures. It has been reported that *Scn1a* mutant mice have the risk for hyperthermia‐induced clonic seizures similar to those observed in patients with DS. Using our heating protocol with an incubator, we evaluated the effects of antiseizure medications on the incidence of hyperthermia‐induced clonic seizures in mutant *Scn1a* knock‐in (KI)/+ mice.

**Methods:**

For clonic seizure induction in *Scn1a* KI/+ mice, each mouse was placed into an incubator (38°C–40°C) and observed for 15 min. Antiseizure medications were administered orally 30 min prior to the experiment. The time to seizure onset and the incidence of clonic seizures were analyzed to evaluate and compare the antiseizure effects among treatment groups.

**Results:**

Our incubation system increased the body temperature of mice and induced clonic seizures in *Scn1a* KI/+ mice. Clobazam (CLB) and valproate (VPA) significantly reduced the incidence of hyperthermia‐induced clonic seizures in *Scn1a* KI/+ mice, with 50% effective dose (ED_50_) values of 1.94 mg/kg (95% confidence interval [CI]: 0.93–3.89 mg/kg) and 255.87 mg/kg (95% CI: 188.66–356.85 mg/kg) respectively, and prolonged the time to seizure onset. In contrast, lamotrigine (LTG), a sodium channel blocker, did not reduce the incidence of hyperthermia‐induced seizures. CLB and LTG did not affect the body temperature. VPA decreased the body temperature compared to the vehicle‐treated mice before and after incubation.

**Conclusion:**

These results reflect clinical findings that CLB and VPA suppress, but LTG does not suppress seizures in patients with DS. Our data demonstrate the validity of our test system to induce hyperthermia‐induced seizures and to evaluate the efficacy of antiseizure medications in *Scn1a* KI/+ mice.

## Abstract 2

Using a stabilized incubator‐based hyperthermia model of Dravet syndrome, we showed that clobazam and valproate significantly reduced heat‐induced clonic seizures and delayed seizure onset in *Scn1a* KI/+ mice. In contrast, lamotrigine showed no protective effect. These findings support the model's translational validity for antiseizure drug screening and therapeutic candidate evaluation.

## Graphical abstract

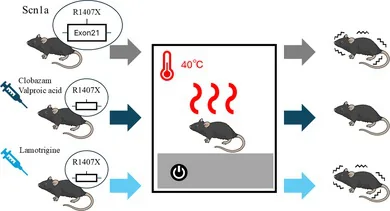

## 1 Introduction

Dravet syndrome (DS) was first described in 1978, when Dravet et al. named the syndrome severe myoclonic epilepsy in infancy [1]. Approximately 80% of patients with DS carry heterozygous loss‐of‐function mutations in the sodium voltage‐gated channel alpha subunit 1 (*SCN1A*) gene [2, 3]. The incidence of DS is estimated to range from 1 in 20 000 to 1 in 40 000 in the US [4]. No clear geographical pattern has been identified [5]. Patients with DS usually develop epileptic tonic–clonic or clonic seizures between 4 and 10 months of age. The initial seizure is often misdiagnosed as a febrile seizure and may not be treated. However, shortly thereafter, within a span of 2 weeks to 2 months, other febrile seizures occur, and afebrile seizures also appear. Additional seizure types appear between 1 and 4 years of age and are often accompanied by developmental delay [1].

Clinically, DS is characterized by drug resistance [6, 7]. Clobazam (CLB) and valproate (VPA) are generally accepted as first‐line antiseizure medications for DS [8]. However, these conventional antiseizure medications are usually insufficient to achieve adequate seizure control in many patients [9]. Lamotrigine (LTG), a sodium channel blocker, is generally not recommended for patients with DS as it has been reported to exacerbate seizures in these individuals [6]. The development of novel antiseizure medications for DS is needed, for which we must establish an efficient screening system to find drug candidates for treatment of DS.

Several DS model mice have been constructed by introducing mutations into the *Scn1a* gene [3]. Ogiwara produced mice carrying an *Scn1a* gene mutation. This knock‐in (KI) mouse line carries a premature stop codon R1407X in exon 21 of mouse *Scn1a*, which is identical to the pathogenic mutation found in three unrelated patients with severe myoclonic epilepsy in infancy (SMEI). SMEI patients and *Scn1a* KI mice have been known to show fever‐sensitive seizures [10, 11]. These *Scn1a* Rx/+ mice have the susceptibility to hyperthermia‐induced seizures, which can be assessed by electroencephalography [12].

Hyperthermia‐induced seizures in mutant *Scn1a* heterozygous mice have been reported using a hot plate [13], heat lamp [14], and hair dryer [15]. There is a need to measure the temperature and adjust the volume of heated air according to body core temperature of mice. These assays have a limited capacity to evaluate a large number of compounds simultaneously.

We aimed to establish a novel experimental method using an incubator and plexiglass chamber to stabilize the ambient temperature and evaluate antiseizure effects on hyperthermia‐induced clonic seizures in DS model mice.

## 2 Methods

### 2.1 Animals

All mouse experiments in the present study were approved by the Animal Care and Use Committee for Otsuka Pharmaceutical Co. Ltd. (approval number: 20‐0175, 21‐0109, 21‐0162, and 21‐0176).

C57BL6/J mice and *Scn1a* KI/+ mice (C57BL/6J background) were provided by Charles River Japan, which was acquired by Jackson Laboratory since 2021. These *Scn1a* KI/+ mice were generated by a CRISPR‐Cas9 system and in vitro fertilization at Charles River Japan. Male mice aged 11–15 weeks were housed in a specific pathogen‐free mouse facility under standard laboratory conditions (12 h light/12 h dark) and had access to food and water ad libitum, except during hyperthermia‐induced seizure experiments.

### 2.2 Drugs

CLB was purchased from Dainippon Sumitomo Pharma Co. Ltd., then extracted from the tablets and purified at Otsuka Pharmaceutical Co. Ltd. VPA was purchased from Fujifilm WAKO Chemicals. LTG was purchased from LKT labs. All drugs were suspended and diluted in vehicle, 5% (w/v) gum arabic (Powdered Acacia, Suzu Pharmaceutical Co Ltd)‐distilled water solution. The vehicle and all drug suspensions were shielded from light and stored at 4°C until use. All drugs were administered orally at a volume of 10 mL/kg. Acute toxicity, except the hypothermia by VPA, was not observed with any drugs at the administered doses.

### 2.3 Measurement of Rectal Temperature

The weighing environment logger and rectal probe were purchased from A&D company (Catalogue No. AD1687).

A rectal probe was inserted into the anus of each mouse to measure rectal temperature before and after incubation of mice.

### 2.4 Induction of Hyperthermia Seizure

**FIGURE 1:**
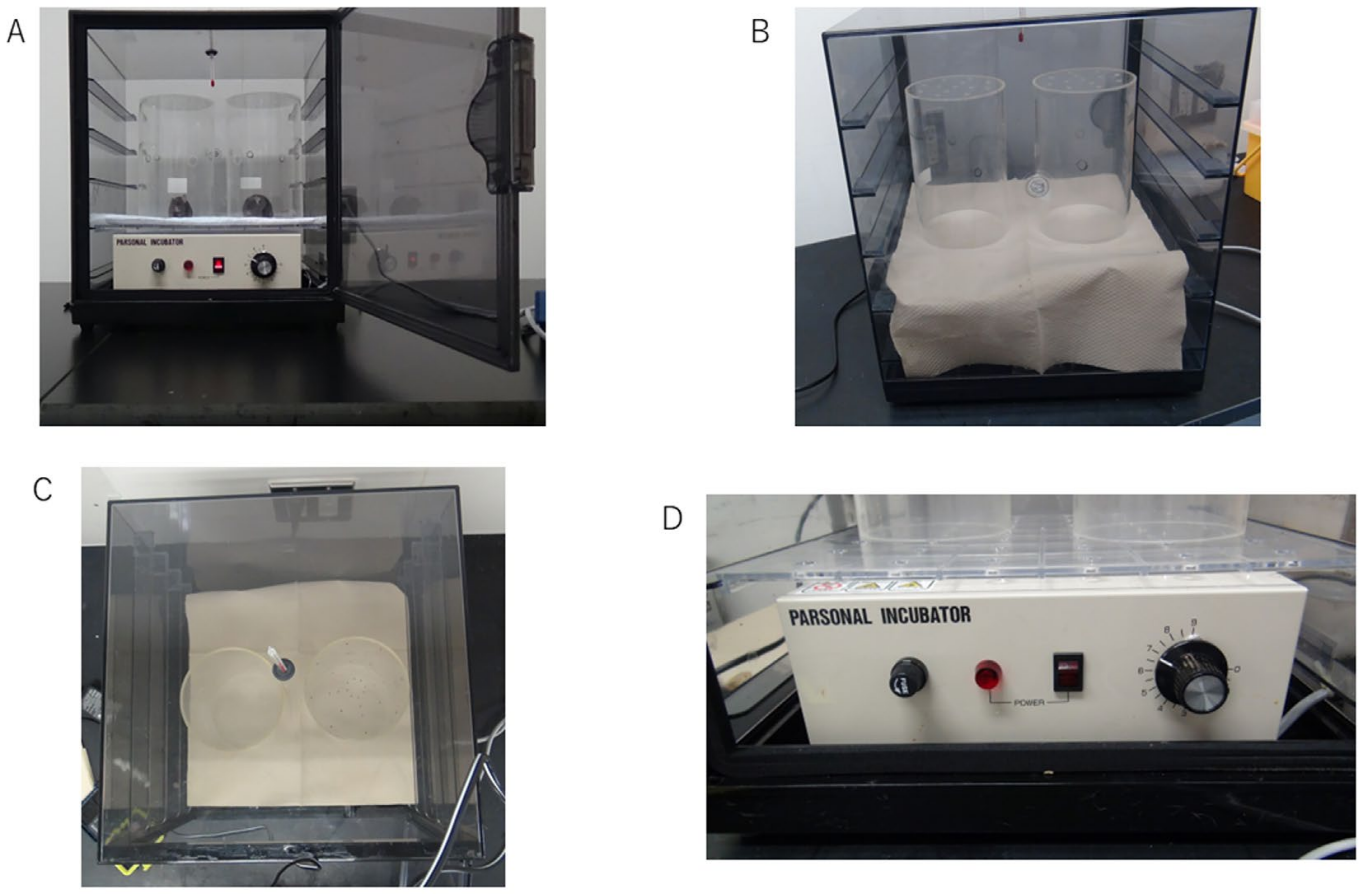
The incubator system to observe the hyperthermia‐induced seizure. This photo shows (A) front, (B) back, (C) top, and (D) close‐up views of our incubator system. The surface of the incubator is transparent. The incubator has a heating element located below the bottom tray. The red switch on the heating element is the power button. The temperature can be adjusted using the dial controller on the heating element. The temperature is monitored by the thermometer on the top of the box. A water‐absorbing sheet is placed above the bottom tray to absorb urine from the mice. The two cylinders are placed on the sheet to observe the two mice simultaneously.

We modified a previously reported method [14]. The hyperthermia‐induced clonic seizure incidence and the time to seizure onset were compared between wild type mice and *Scn1a* KI/+ mice. To elevate the body temperature, each mouse was put into an incubator (AS ONE corporation, Catalogue No. UI‐50, Figure 1) at 38°–40°C and observed for seizures for 900 s. The alcohol thermometer (SATO KEIRYOKI MFG. Co Ltd., Catalogue No. 0600‐00) was set on the top of the incubator to check the temperature in the incubator. The time to seizure onset was measured by an observer. The *Scn1a* KI/+ mice were assigned to groups based on the time to seizure onset.

*Scn1a* KI/+ mice were weighed before the first administration of the drugs. Each mouse was orally administered vehicle or drugs at a volume of 10 mL/kg of body weight 30 min before incubation. Then, each mouse was put into an incubator at 38°C–40°C to elevate the body temperature and was observed for seizures for 900 s. The rectal temperature was measured before and after incubation. The time to seizure onset was measured by an observer. Hyperthermia induced by incubation was confirmed in all mice. The observer was blinded to the treatments received by each mouse.

### 2.5 Statistical Analysis

All statistical analyses were performed using SAS Software (Release 9.4, SAS Institute Inc.). The significance level was set at 0.05 (two‐sided).

The difference between the wild type group and the *Scn1a* KI/+ group was assessed by the Fisher exact probability test for the clonic seizure incidence, and the log‐rank test for the time to seizure onset.

We used Kaplan–Meier analysis on hyperthermia‐induced clonic seizures to compare the effect of antiseizure medications. Kaplan–Meier analysis on seizure free rate was reported clinically [16] The difference between vehicle group and each dose group of test drugs was assessed by the Kaplan–Meier plot and log‐rank test with Dunnett's adjustment for multiplicity for the time to seizure onset. The 50% effective dose (ED_50_) value and 95% confidence interval (95% CI) of drugs in preventing clonic seizure incidence were calculated by probit analysis using the seizure rates from all treatment groups.

The difference in pre‐ and post‐incubation body temperatures was analyzed with a paired *t*‐test. The difference in body temperature between the vehicle group and the compound group at each time point, before and after incubation, was analyzed using two‐way repeated measures ANOVA. The relationship between body temperature metrics and the time to seizure onset was analyzed using Spearman coefficient correlation.

## 3 Results

### 3.1 Hyperthermia‐Induced Seizure in *Scn1a* Mutant Mice

**FIGURE 2:**
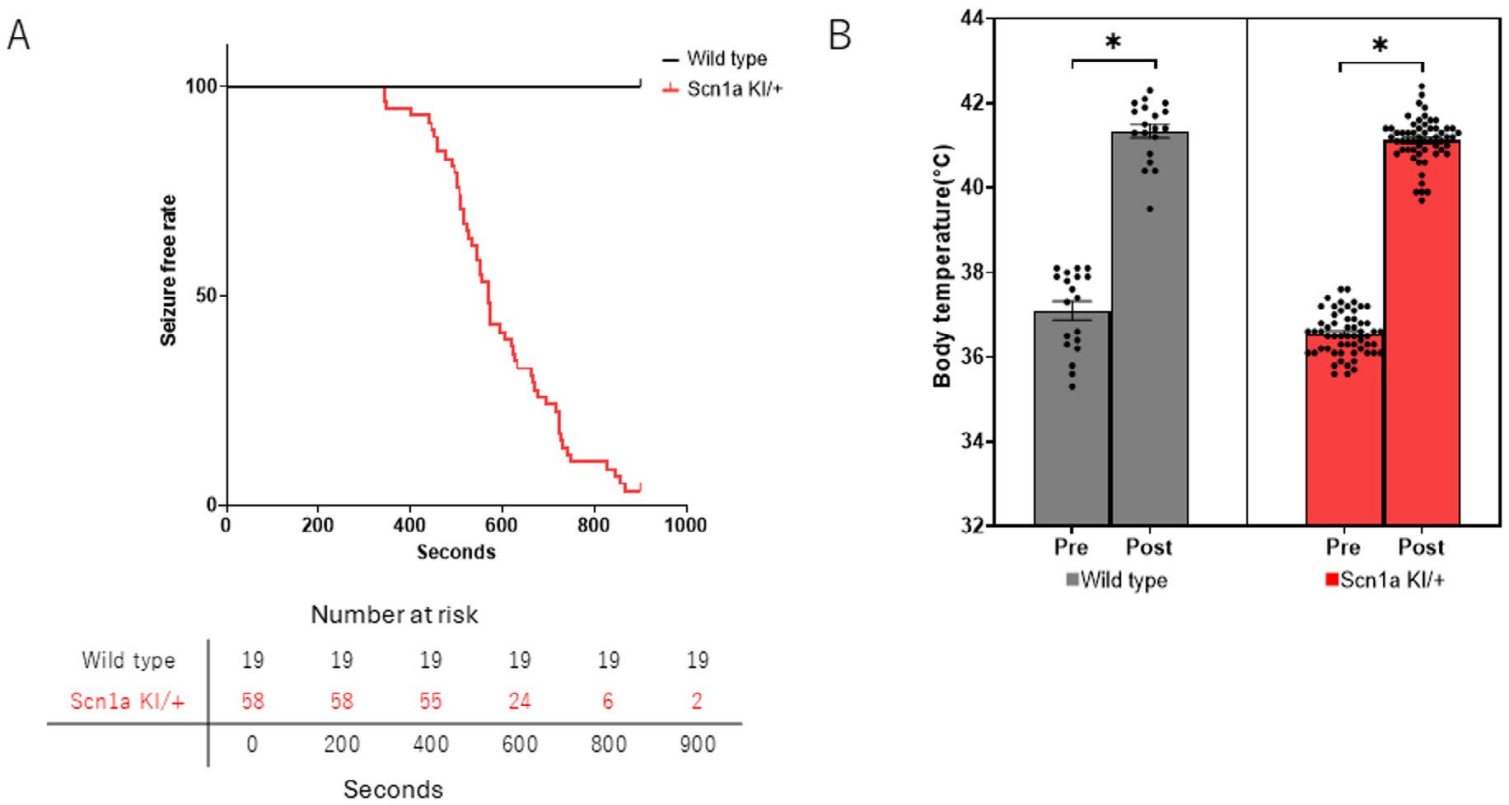
The difference in the time to seizure onset and body temperature between wild type and *Scn1a* KI/+. (A) The time to seizure onset in wild type and *Scn1a* KI/+ mice is shown as a Kaplan–Meier plot. (B) Body temperature was measured before and after incubation. Mice were incubated and observed for seizures for 900 s (wild type mice: *n* = 19, *Scn1a* KI/+ mice: *n* = 58). Data in (A) were analyzed using log‐rank test (*p* < 0.001). Data in (B) were presented as mean ± SEM. *p < 0.05 versus pre‐incubation (paired *t*‐test).

The results of the comparison between wild type mice and *Scn1a* KI/+ mice are shown in Table 1 and Figure 2A. The majority of *Scn1a* KI/+ mice showed hyperthermia‐induced seizures. None of the wild type mice showed hyperthermia‐induced seizures. Following incubation, the body temperature of wild type mice increased from 37.9°C to 40.8°C (*p* < 0.05, paired *t*‐test), while that of *Scn1a* KI/+ mice rose from 36.6°C to 40.9°C (*p* < 0.05, paired *t*‐test) (Figure 2B).

**TABLE 1:** The difference in clonic seizure incidence between wild type and *Scn1a* KI/+ mice.

Genotype	*n*/group	The number of mice showing clonic seizure
Wild type	19	0
*Scn1a* KI/+	58	56

*Note:* Each mouse was placed in our incubator system at 38°C–40°C and observed for seizures for 900 s. Wild type mice did not show seizures. In contrast, almost all *Scn1a* KI/+ mice exhibited seizures. The difference between the wild type group and the *Scn1a* KI/+ group was assessed by Fisher exact probability test for the clonic seizure incidence. The *p*‐value was < 0.001.

### 3.2 Evaluation of Antiseizure Effects of Drugs on Hyperthermia‐Induced Seizure

**FIGURE 3:**
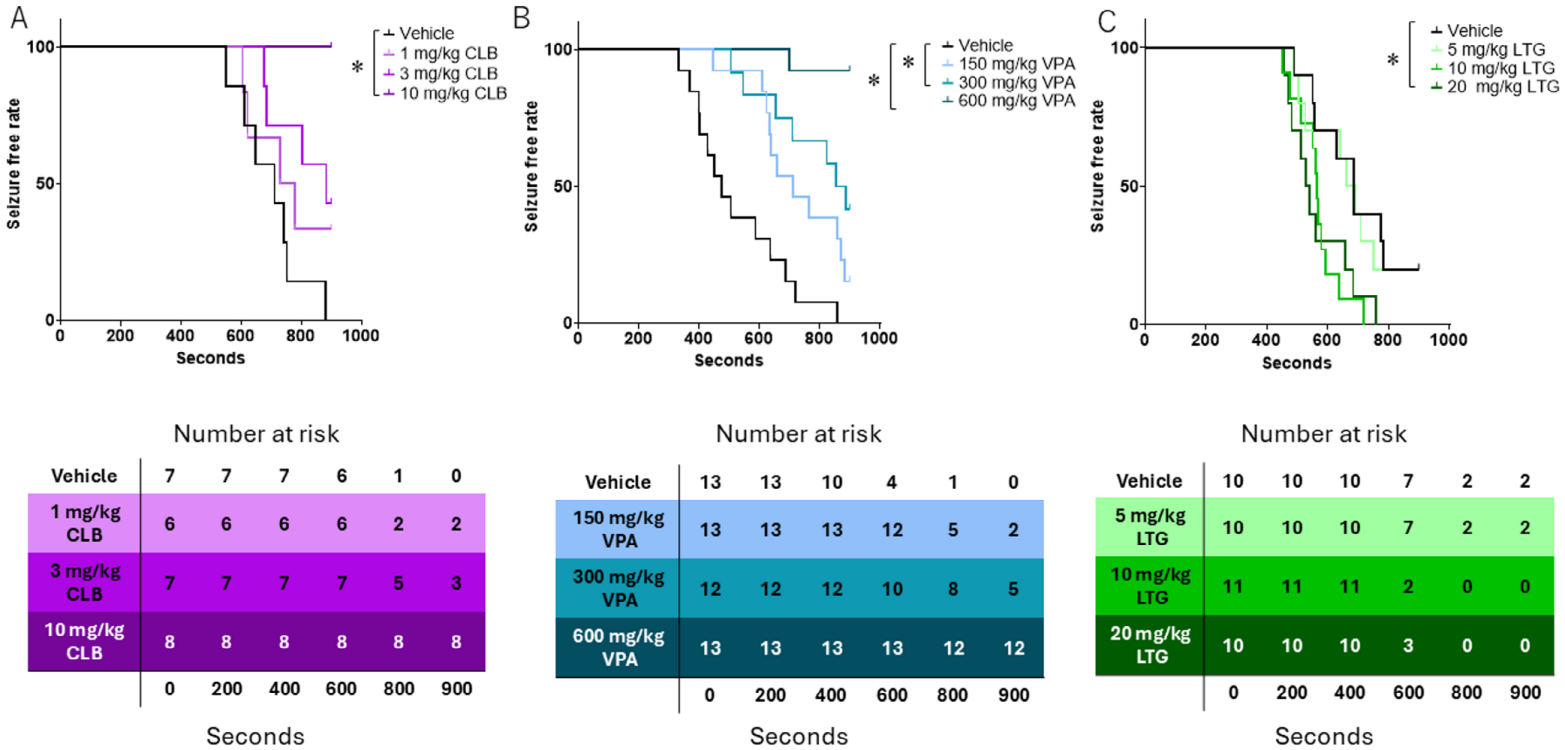
Effects of antiseizure medications on the hyperthermia‐induced seizures in *Scn1a* KI/+ mice. Animals were incubated and observed for seizures for 15 min with the 30‐min pretreatment with antiseizure medications. The time to seizure onset was shown as a Kaplan–Meier plot. Probability indicates the ratio of animals not showing hyperthermia‐induced seizures. (A) CLB (0, 1, 3, 10 mg/kg) groups and (B) VPA (0, 150, 300, 600 mg/kg) groups were evaluated for the seizure onset. The time to seizure onset in CLB and VPA groups was longer than that in vehicle group. The time‐to‐event curves were compared using the log‐rank test with Dunnett's adjustment versus the vehicle group. *p* = 0.31, 0.13, and < 0.001 for CLB (1, 3, and 10 mg/kg, respectively); *p* = 0.14, 0.002, and < 0.001 for VPA (150, 300 and 600 mg/kg, respectively). The number of samples in each group was: CLB 0 mg/kg (*n* = 7), CLB 1 mg/kg (*n* = 6), CLB 3 mg/kg (*n* = 7), CLB 10 mg/kg (*n* = 8), VPA 0 mg/kg (*n* = 13), VPA 150 mg/kg (*n* = 13), VPA 300 mg/kg (*n* = 12), VPA 600 mg/kg (*n* = 13). (C) LTG (0, 5, 10, 20 mg/kg) groups were evaluated for the onset. The number of samples in each group was: LTG 0 mg/kg (*n* = 10), LTG 5 mg/kg (*n* = 10), LTG 10 mg/kg (*n* = 11), LTG 20 mg/kg (*n* = 10). The time‐to‐event curves were compared using the log‐rank test with Dunnett's adjustment versus the vehicle group. *p* = 0.98, 0.051, and 0.050 for LTG (5, 10, and 20 mg/kg, respectively) *p < 0.05 versus pre‐incubation (Log‐rank test).

The incidence of hyperthermia‐induced clonic seizures when the mice were treated with CLB, VPA, or LTG is shown in Figure 3. In Figure 3A, CLB dose‐dependently delayed seizure onset. The log‐rank test with Dunnett's adjustment revealed a significant prolongation in the time to seizure onset with 10 mg/kg CLB treatment (*p* < 0.001) compared with the vehicle group. In Figure 3B, VPA dose‐dependently suppressed the seizure onset, with significant prolongations of seizure latency observed at 300 and 600 mg/kg VPA treatments (*p* = 0.002 and *p* < 0.001, respectively). Figure 3C shows that LTG did not suppress hyperthermia‐induced seizures. The 10, 20 mg/kg doses of LTG tended to shorten the time to seizure onset (*p* = 0.051, *p* = 0.050, respectively).

**FIGURE 4:**
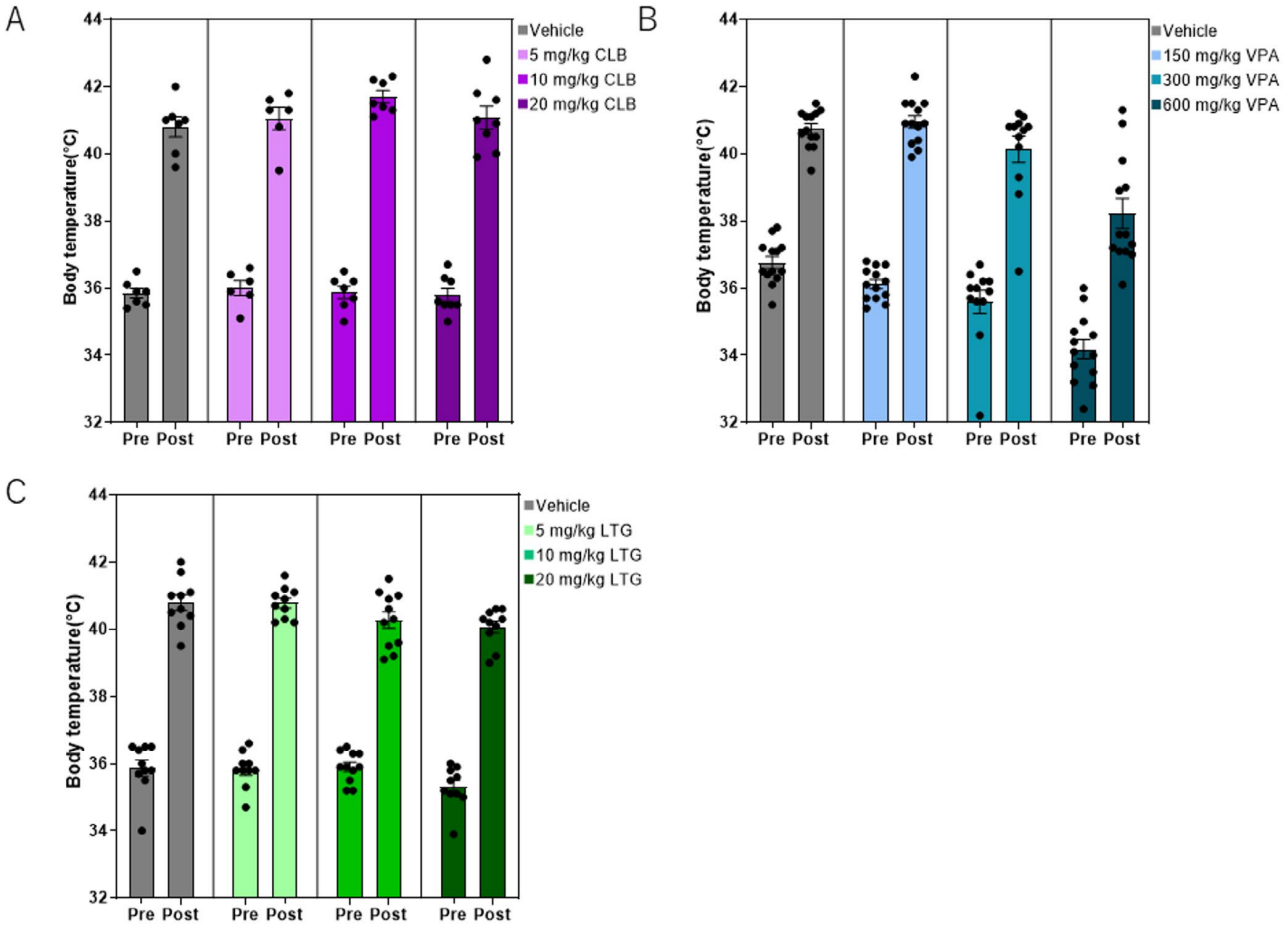
Effects of antiseizure medications on the body temperature. The body temperature of mice was measured 30 min after the treatment with antiseizure medications. Each mouse was incubated for 900 s. The body temperature of *Scn1a* KI/+ mice was measured just after incubation. (A, C) The body temperature in CLB (1, 3, 10 mg/kg) groups and LTG (5, 10, 20 mg/kg) groups was similar to the vehicle group before and after incubation. (B) The body temperature in VPA (150, 300, 600 mg/kg) groups decreased dose dependently before and after incubation. The number of samples in each group was: CLB 0 mg/kg (*n* = 7), CLB 1 mg/kg (*n* = 6), CLB 3 mg/kg (*n* = 7), CLB 10 mg/kg (*n* = 8), VPA 0 mg/kg (*n* = 13), VPA 150 mg/kg (*n* = 13), VPA 300 mg/kg (*n* = 12), VPA 600 mg/kg (*n* = 13), LTG 0 mg/kg (*n* = 10), LTG 5 mg/kg (*n* = 10), LTG 10 mg/kg (*n* = 11), LTG 20 mg/kg (*n* = 10). These data are presented as mean ± SEM.

The rectal temperature was assessed before and after incubation. Figure 4 and Figure S1 show that the treatment with neither CLB nor LTG had a significant effect on the body temperature. In contrast, the treatment with VPA significantly lowered the body temperature of mice compared to the vehicle treatment both before and after incubation (*p* < 0.05, Two‐way repeated measures ANOVA, <Pre‐Post and Dose>). Figure S2 shows the relationship between body temperature metrics and the time to seizure onset. Spearman correlation coefficients were computed. (CLB; *r* = −0.02, *p* = 0.92, VPA; *r* = −0.28, *p* = 0.050, LTG; *r* = 0.58, *p* < 0.01).

**FIGURE 5:**
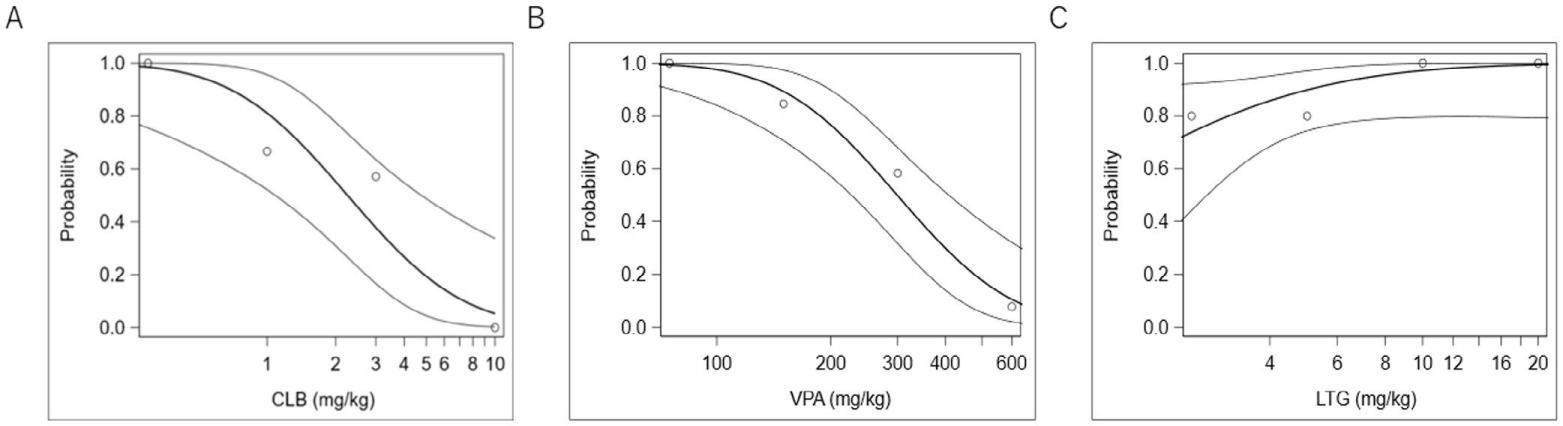
Probit analysis on the effects of antiseizure medications. Animals were incubated and observed for seizures for 15 min with the 30‐min pretreatment with antiseizure medications. ED_50_ values and 95% CIs of each drug were determined using probit analysis. (A) CLB (0, 1, 3, 10 mg/kg), (B) VPA (0, 150, 300, 600 mg/kg), and (C) LTG (0, 5, 10, 20 mg/kg) groups were evaluated. ED50 value of LTG was not calculated because LTG did not reduce seizure incidence. The number of samples in each group was: CLB 0 mg/kg (*n* = 7), CLB 1 mg/kg (*n* = 6), CLB 3 mg/kg (*n* = 7), CLB 10 mg/kg (*n* = 8), VPA 0 mg/kg (*n* = 13), VPA 150 mg/kg (*n* = 13), VPA 300 mg/kg (*n* = 12), VPA 600 mg/kg (*n* = 13), LTG 0 mg/kg (*n* = 10), LTG 5 mg/kg (*n* = 10), LTG 10 mg/kg (*n* = 11), LTG 20 mg/kg (*n* = 10).

Figure 5 and Table 2 show that the ED_50_ values and 95% CIs of CLB and VPA in preventing clonic seizure incidence were calculated by probit analysis using the seizure rates from all treatment groups obtained at the time when the last mouse in the vehicle group showed seizure.

**TABLE 2:** Effect of CLB and VPA on the development of hyperthermia‐induced seizure in *Scn1a* KI/+ mice.

Drug	ED_50_	95% CI
CLB	1.94 mg/kg	0.93–3.89
VPA	255.87 mg/kg	188.66–356.85
LTG	—	—

*Note:* The ED_50_ values and 95% confidence intervals of the inhibitory effect of CLB and VPA against hyperthermia‐induced seizures were calculated using probit analysis. Each mouse was placed in our incubator system at 38°C–40°C after the treatment with each compound. Each mouse was observed for seizures for 900 s.

## 4 Discussion

Firstly, wild type mice and *Scn1a* KI/+ mice were put into an incubator and observed for seizures. The majority of *Scn1a* KI/+ mice showed hyperthermia‐induced seizure, but almost all of the wild type mice did not. Secondly, *Scn1a* KI/+ mice were put into an incubator and observed for seizures 30 min after pretreatment with vehicle or a test drug. CLB and VPA significantly reduced the incidence of hyperthermia‐induced clonic seizures and prolonged the time to seizure onset. In contrast, LTG did not reduce the incidence or prolong the time to seizure onset. VPA induced hypothermia in DS model mice.

We confirmed that treatment of some antiseizure medications reduced seizures of *Scn1a* KI/+ mice. These results suggest that this incubator system enables us to evaluate the effect of antiseizure medications on DS mice model effectively. No wild type mice showed hyperthermia‐induced clonic seizure, while almost all of *Scn1a* KI/+ mice did at approximately 41°C body temperature. This suggests that gene mutation of *Scn1a* does not affect the thermoregulatory function but causes the febrile seizure. This incubation system induced hyperthermia‐induced clonic seizure in only DS model mice.

First‐line management involves CLB and VPA [17]. These drugs significantly suppressed the incidence of hyperthermia‐induced clonic seizures in *Scn1a* KI/+ mice like in humans. CLB was the most effective drug for seizure reduction in the *Scn1a* KI/+ mice, with a protective benefit against the thermally induced seizures. This is consistent with the clinical experience from DS patients, in whom CLB treatment resulted in seizure reduction in almost 75% of cases, and is the most consistently beneficial treatment reported in the literature [13]. Previous studies with DS model mice carrying mutant *Scn1a* gene reported beneficial effects with benzodiazepines, including CLB and clonazepam, by the assessment of electroencephalography [10, 12]. We confirmed that this incubation system induced hyperthermia, and CLB suppressed the hyperthermia‐induced clonic seizure. Efficacy of CLB (1.5–2.0 mg/kg) was reported clinically [18]. Our incubation model and following statistical analysis predicted effective dose.

VPA reduced the seizure rate and induced hypothermia in DS model mice. This hypothermia observed in mice may reflect the cool side effects of VPA administration in clinical settings, although the mechanism underlying VPA‐induced thermoregulatory disturbance remains unclear [19]. The hypothermia induced by VPA treatment may have blocked body temperature dependent induction of clonic seizure.

LTG had neither hypothermia nor antiseizure effect on hyperthermia‐induced seizure in DS model mice. It has been reported in humans that antiseizure medications with sodium channel blocking activity, such as LTG, have a risk to aggravate DS conditions, and hence are generally avoided in patients with DS [20].

In the present study, we demonstrated that hyperthermia induced seizures in the majority of *Scn1a* KI/+ mice in this system, and the treatment with CLB and VPA markedly reduced the clonic seizure incidence and prolonged the time to seizure onset in the DS mice, indicating prevention of the hyperthermia‐induced seizure by these drugs which are clinically used for treatment of DS patients. We have established the drug screening system utilizing an incubator and a plexiglass chamber to evaluate the antiseizure effects of compounds on hyperthermia‐induced clonic seizures in DS model mice.

## Author Contributions

F.S., N.A., Y.Y., K.M., and T.F. were involved in the conception and design of the experiments. F.S., T.W., N.J., M.N., M.O., Y.M., N.A., and Y.Y. performed the experiments. F.S. and Y.K. performed the statistical analyses. F.S. and N.A. wrote the manuscript. All authors reviewed and approved the final manuscripts.

## Funding

This work was supported by Otsuka Pharmaceutical Co. Ltd.

## Ethics Statement

All experimental procedures were approved by Otsuka Pharmaceutical Co. Ltd. No human study was performed. All animal care and experimental procedures were carried out in accordance with “Guidelines for Animal Care and Use in Otsuka Pharmaceutical Co. Ltd.” (approval number 20‐0175, 21‐0109, 21‐0162, 21‐0176).

## Consent

The authors have nothing to report.

## Conflicts of Interest

All the authors are employees and own stock of Otsuka Pharmaceutical Co. Ltd.

## Supporting information


**Figure S1:** Body temperature was measured before (Pre) and after (Post) incubation. The Post/Pre ratio of body temperature is shown in panels A–C, and the change in body temperature (Δ*T*; Post − Pre) is shown in panels D–F. (A, D) CLB, (B, E) VPA, and (C, F) LTG. Data are presented as mean ± SEM. The Post/Pre ratio and Δ*T* were analyzed separately using one‐way ANOVA with dose as the factor. When appropriate, Dunnett's multiple‐comparison test was used to compare each dose group with the corresponding vehicle group. For CLB, no significant dose effect was observed for the Post/Pre ratio [*F*(3, 24) = 1.67, *p* = 0.201] or Δ*T* [*F*(3, 24) = 1.74, *p* = 0.1848]. For VPA, significant dose effects were observed for the Post/Pre ratio [*F*(3, 47) = 3.12, *p* = 0.0347] and Δ*T* [*F*(3, 47) = 3.39, *p* = 0.0254]. Dunnett‐adjusted comparisons with the vehicle group showed significant differences for VPA 150 mg/kg for both the Post/Pre ratio (adjusted *p* = 0.0176) and Δ*T* (adjusted *p* = 0.0246), whereas the other dose groups were not significantly different from the vehicle group. For LTG, no significant dose effect was observed for the Post/Pre ratio [*F*(3, 37) = 1.18, *p* = 0.3294] or Δ*T* [*F*(3, 37) = 1.27, *p* = 0.2988]. The numbers of animals in each group were as follows: CLB, 0 mg/kg (*n* = 7), 1 mg/kg (*n* = 6), 3 mg/kg (*n* = 7), and 10 mg/kg (*n* = 8); VPA, 0 mg/kg (*n* = 13), 150 mg/kg (*n* = 13), 300 mg/kg (*n* = 12), and 600 mg/kg (*n* = 13); and LTG, 0 mg/kg (*n* = 10), 5 mg/kg (*n* = 10), 10 mg/kg (*n* = 11), and 20 mg/kg (*n* = 10). Adjusted *p* values from Dunnett's multiple‐comparison test are indicated in the figure.

**Figure S2:** Effects of antiseizure medications on body temperature measured before and after incubation. The relationship between post incubation body temperature and the time to seizure onset. (A) CLB (0, 1, 3, 10 mg/kg), (B) VPA (0, 150, 300, 600 mg/kg), and(C) LTG (0, 5, 10, 20 mg/kg) groups were evaluated. The number of samples in each group was: CLB 0 mg/kg (*n* = 7), CLB 1 mg/kg (*n* = 6), CLB 3 mg/kg (*n* = 7), CLB 10 mg/kg (*n* = 8), VPA 0 mg/kg (*n* = 13), VPA 150 mg/kg (*n* = 13), VPA 300 mg/kg (*n* = 12), VPA 600 mg/kg (*n* = 13), LTG 0 mg/kg (*n* = 10), LTG 5 mg/kg (*n* = 10), LTG 10 mg/kg (*n* = 11), LTG 20 mg/kg (*n* = 10).

## Data Availability

The data presented in this study are available from the corresponding author upon request.
